# Clinicopathological characteristic and clinical handling of the patients with 2 cm or less gastric GISTs

**DOI:** 10.1186/2193-1801-2-469

**Published:** 2013-09-17

**Authors:** Mikinori Kataoka, Takashi Kawai, Hidekazu Ikemiyagi, Takashi Fujii, Mari Fukuzawa, Masakatsu Fukuzawa, Keisuke Kubota, Masashi Yoshida, Shinji Suzuki, Masaki Kitajima

**Affiliations:** Department of Gastroenterology and Hepatology, International University Of Health and Welfare Mita Hospital, 1-4-3 Mita, Minatoku, Tokyo, 108-8329 Japan; Endoscopy Center, Tokyo Medical University Hospital, Tokyo, Japan; Department of Gastroenterology and Hepatology, Tokyo Medical University Hospital, Tokyo, Japan; Department of Gastroenterological Surgery, International University Of Health and Welfare Mita Hospital, Tokyo, Japan

**Keywords:** Submucosal tumors, Gastrointestinal stromal tumors, Endoscopic mucosal cutting biopsy

## Abstract

**Background:**

We previously reported that safety and efficacy of mucosal cutting biopsy for diagnosing included 2 cm or less gastric GISTs. However, there have been no reports stating the clinicopathological characteristic and clinical handling of the patients with 2 cm or less gastric GISTs. The aim of our study is to investigate the clinicopathological characteristic and clinical handling of the patients with 2 cm or less gastric GISTs.

**Methods:**

The 19 patients diagnosed with GIST by mucosal cutting biopsy were divided into 2 groups: Group I; subjects were GISTs with 2 cm or less, Group II; subjects were GISTs >2 cm. We compared the 2 groups in terms of mean age, tumor size, tumor site, histopathological risk grade. In cases that underwent surgery with a diagnosis of GIST, we compared the pre- and postoperative histopathological diagnosis, and the histopathlogical risk grade within each group.

**Results:**

The mean age and tumor size were significantly higher in Group I than in Group II. Meanwhile, there were no significant differences between the 2 groups, sex ratio, tumor site. All lesions were at histopathological risk grade at very low risk and low risk respectively. In 17 patients with GIST who underwent surgery, the histopathological diagnoses, immunostaining were in agreement with those from the mucosal cutting biopsy specimens in all cases, but mitotic count of one patient was not in agreement in group II.

**Conclusions:**

The 2 cm or less gastric GISTs diagnosed with histpathlogical very low risk can be considered acceptable to follow-up.

## Introduction

Gastrointestinal stromal tumors(GISTs) are the most common submucosal tumors(SMTs) in the gastrointestinal(GI) tract. The majority of GISTs are located in the stomach, followed by the small intestine (20%-30%), large intestine (5%), and esophagus (1%) (Fletcher et al. 2002a; Kitamura et al. 2003). GISTs show a wide variety of clinical behavior, from benign to frankly malignant, and outcome in individual patients remains difficult to predict (Grotz & Donohue 2011). The Japanese GIST Therapeutic Guidelines (GIST 2008) published in March 2008 state that important parameters of malignancy and prognosis are thought to be growing tumor size, surface morphological change, histopathological mitotic and cellularity proliferative index. And surgical excision is indicated if a histopathological diagnosis of GIST is made. However, as with other SMTs, establishing a histologic diagnosis has been considered extremely difficult. So as it stands now, the patients with 2 cm or less gastric SMTs have been followed up. We previously reported that safety and efficacy of mucosal cutting biopsy for diagnosing included 2 cm or less gastric GISTs (Kataoka et al. 2013).

The aim of our study is to investigate the clinicopathological characteristic and clinical handling of the patients with 2 cm or less gastric GISTs.

## Subjects and methods

Of a total of 29 patients without symptom diagnosed with submucosal tumors emerged from the muscular layer by endoscopic ultrasound (EUS) who underwent mucosal cutting biopsy between September 2008 and December 2011(Table 1), we diagnosed 19 patients (14 men and 5 women) histopathologically as GIST. The 19 patients were divided into 2 groups: Group I; subjects were GISTs with 2 cm or less, Group II; subjects were GISTs with >2 cm. We compared the 2 groups in terms of mean age, tumor size, tumor site (lower third: L; middle third: M; upper third: U), and histopathological risk grade according to Fletch’s criteria (Fletcher et al. 2002b), mitotic and cellularity proliferative index. In cases that proceeded to surgical resection with a diagnosis of GIST, we compared the histopathological diagnoses from the mucosal cutting biopsy specimens and surgically resected specimens within each group. Specimens were evaluated using hematoxylin-eosin (HE) staining, as well as staining for c-kit, CD34, α-SMA, S-100 protein, the cellular proliferation antigen Ki-67, and mitotic counts (per 50 high power fields).

**Table 1:** **The characteristics of the 29 patients with mucosal cutting biopsy**

							Endoscopic finding		
Case no.	Gender	Age (yrs)	Location (L/M/U)	Size (mm)	Histopathological findings of biopsy specimens	EUS appearance	Smooth mucosa	Mucosal ulceration	Umbilication
1	M	37	L	20	GIST	Homogeneous	+	−	−
2	M	66	U	40	GIST	Homogeneous	+	−	−
3	M	61	U	30	GIST	Homogeneous	+	−	−
4	M	27	M	18	Heterotropic pancreas	Homogeneous	+	−	−
5	M	75	M	15	GIST	Homogeneous	+	−	−
6	M	58	U	20	GIST	Homogeneous	+	−	−
7	F	51	M	8	Leiomyoma	Homogeneous	+	−	−
8	F	85	M	30	GIST	Homogeneous	+	−	−
9	M	36	U	30	Heterotropic pancreas	Homogeneous	+	−	−
10	M	50	L	20	Leiomyoma	Homogeneous	+	−	−
11	M	72	U	25	GIST	Homogeneous	+	−	−
12	F	74	L	15	GIST	Homogeneous	+	−	−
13	M	50	U	15	GIST	Homogeneous	+	−	−
14	F	68	U	12	Leiomyoma	Homogeneous	+	−	−
15	F	68	U	19	GIST	Homogeneous	+	−	−
16	M	46	L	10	GIST	Homogeneous	+	−	−
17	F	49	U	20	GIST	Homogeneous	+	−	−
18	M	57	L	20	GIST	Homogeneous	+	−	−
19	F	46	M	30	Heterotropic pancreas	Homogeneous	+	−	−
20	M	57	M	30	GIST	Homogeneous	+	−	−
21	F	75	M	20	GIST	Homogeneous	+	−	−
22	F	64	U	20	Leiomyoma	Homogeneous	+	−	−
23	M	47	U	30	Leiomyoma	Homogeneous	+	−	−
24	F	54	L	20	Leiomyoma	Homogeneous	+	−	−
25	F	33	M	18	Leiomyoma	Homogeneous	+	−	−
26	M	73	U	25	GIST	Homogeneous	+	−	−
27	M	68	U	25	GIST	Homogeneous	+	−	−
28	M	42	L	18	GIST	Homogeneous	+	−	−
29	M	69	M	25	GIST	Homogeneous	+	−	−

*EUS*: endoscopic ultrasonagraphy.

*α-SMA*: α-smooth muscle actin.

*L*: lower third; *M*: middle third; *U*: upper third.

Statistical analysis was performed using the Fisher exact test. A *p*-value of less than 0.05 was considered to represent a statistically significant difference.

In this study, the method of histologic diagnosis for gastric GISTs was approved by the Institutional Review Board of Tokyo Medical University.

## Results

Table 2 shows the characteristics of the patients in both groups. The mean age was significantly higher in Group I than in Group II (*p <* 0.05). Naturally, mean tumor size was also significantly higher in Group I than in Group II (*p <* 0.05). Meanwhile, there were no significant differences in sex ratio, tumor site. However, in tumor site, GISTs >2 cm were not on L site but on M and U sites. The mitotic count of 11 GIST lesions in Group I and 8 of GIST lesions in Group II were all fewer than 5 per 50 HPF and all lesions were at histopathological risk grade at very low risk (11/11, 100%) and low risk (8/8, 100%) respectively, according to Fletch’s criteria

**Table 2:** **Patient characteristics of the 2 groups**

	Group I	Group II
	(n=11)	(n=8)
**Mean age (years)**	57.3±13.8(37–75)*	68.9±8.4(57–85)
**Sex ratio**	7:4	7:1
**Mean tumor size (mm)**	17.4±3.2(10–20)*	28.7±5.1(25–40)
**Tumor size**	L5 M2 U4	L0 M3 U5
**Histological risk grade** ^**+**^	very low risk (11/11;100%)	low risk (8/8;100%)

Values are presented as means ± standard deviation (SD).

Group I, patients with GISTs smaller tahn 2 cm; Group II, patients with GISTs>2 cm.

*p<0.05 compared with Group II.

+: Fletch’s criteria.

Of the 19 patients given a diagnosis of GIST, one patient from each group refused surgical treatment owing to advanced age and the remaining 17 patients underwent surgical resection of their tumor. The histopathological, immunostaining and mitotic count findings from the surgically resected specimens were in agreement with those from the mucosal cutting biopsy specimens in all 11 cases in Group I. On the other hand in Group II, histopathological and immunostaining findings were also in agreement, while mitotic count of one patient was not in agreement. Therefore, the histopathlogical risk grade of one case changed to moderate risk after surgically resected specimen (Table 3).

**Table 3:** **Comparison of findings from mucosal cutting biopsy specimens and surgically resected specimens**

Group I	Case		CD34	c-kit	α-SMA	Desmin	s-100	Ki-67	Nuclear fission
	**1**	**Biopsy**	**+**	**+**	**−**	**−**	**−**	**1%**	**−**
		**Resected specimen**	**+**	**+**	**−**	**−**	**−**	**1%**	**−**
	**2**	**Biopsy**	**+**	**+**	**−**	**−**	**−**	**<10%**	**−**
		**Resected specimen**	**+**	**+**	**−**	**−**	**−**	**1%**	**2**
	**3**	**Biopsy**	**+**	**+**	**−**	**−**	**−**	**1%**	**−**
		**Resected specimen**	**+**	**+**	**−**	**−**	**−**	**1%**	**<5**
	**4**	**Biopsy**	**+**	**+**	**−**	**−**	**−**	**1%**	**−**
		**Resected specimen**	**+**	**+**	**−**	**−**	**−**	**<1%**	**−**
	**5**	**Biopsy**	**+**	**+**	**−**	**−**	**−**	**2%**	**−**
		**Resected specimen**	**+**	**+**	**−**	**−**	**−**	**1%**	**−**
	**6**	**Biopsy**	**+**	**+**	**−**	**−**	**−**	**3%**	**−**
		**Resected specimen**	**+**	**+**	**−**	**−**	**−**	**3%**	**−**
	**7**	**Biopsy**	**+**	**+**	**−**	**−**	**−**	**<10%**	**<5**
		**Resected specimen**	**+**	**+**	**−**	**−**	**−**	**<10%**	**<5**
	**8**	**Biopsy**	**+**	**+**	**−**	**−**	**−**	**<10%**	**−**
		**Resected specimen**	**+**	**+**	**−**	**−**	**−**	**5%**	**−**
	**9**	**Biopsy**	**+**	**+**	**−**	**−**	**−**	**<10%**	**<5**
		**Resected specimen**	**+**	**+**	**−**	**−**	**−**	**<10%**	**<5**
	**10**	**Biopsy**	**+**	**+**	**−**	**−**	**−**	**<10%**	**−**
		**Resected specimen**	**+**	**+**	**−**	**−**	**−**	**<10%**	**−**
**Group II**	**Case**		**CD34**	**c-kit**	**α-SMA**	**Desmin**	**s-100**	**Ki-67**	**nuclear fission**
	**1**	**Biopsy**	**+**	**+**	**−**	**−**	**−**	**<10%**	**−**
		**Resected specimen**	**+**	**+**	**−**	**−**	**−**	**<3%**	**<3**
	**2**	**Biopsy**	**+**	**+**	**−**	**−**	**−**	**<10%**	**<5**
		**Resected specimen**	**+**	**+**	**−**	**−**	**−**	**3%**	**4**
	**3**	**Biopsy**	**+**	**+**	**−**	**−**	**−**	**3%**	**−**
		**Resected specimen**	**+**	**+**	**−**	**−**	**−**	**3%**	**8**
	**4**	**Biopsy**	**+**	**+**	**−**	**−**	**−**	**<1%**	**−**
		**Resected specimen**	**+**	**+**	**−**	**−**	**−**	**<5%**	**−**
	**5**	**Biopsy**	**+**	**+**	**−**	**−**	**−**	**3%**	**−**
		**Resected specimen**	**+**	**+**	**−**	**−**	**−**	**3%**	**−**
	**6**	**Biopsy**	**+**	**+**	**−**	**−**	**−**	**<10%**	**−**
*α-SMA*:		**Resected specimen**	**+**	**+**	**−**	**−**	**−**	**<5%**	**<3**
*α*-smooth muscle actin	**7**	**Biopsy**	**+**	**+**	**−**	**−**	**−**	**<10%**	**−**
		**Resected specimen**	**+**	**+**	**−**	**−**	**−**	**3%**	**−**

All surgical cases and follow-up cases were confirmed the healing of wound by surgical specimens and endoscopy. And there were no cases that had rapid growth in follow-up cases.

### Case 1

**Figure 1:**
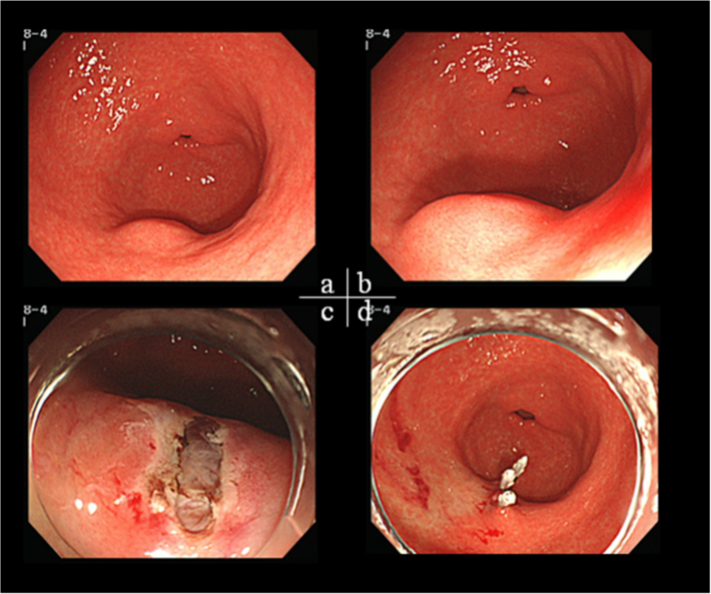
**Mucosal cutting biopsy. a**,**b)** A 10-mm SMT on the greater curvature of the antrum **c)** Mucosal opening was made by mucosal cutting **d)** Wound closure with clips.

**Figure 2:**
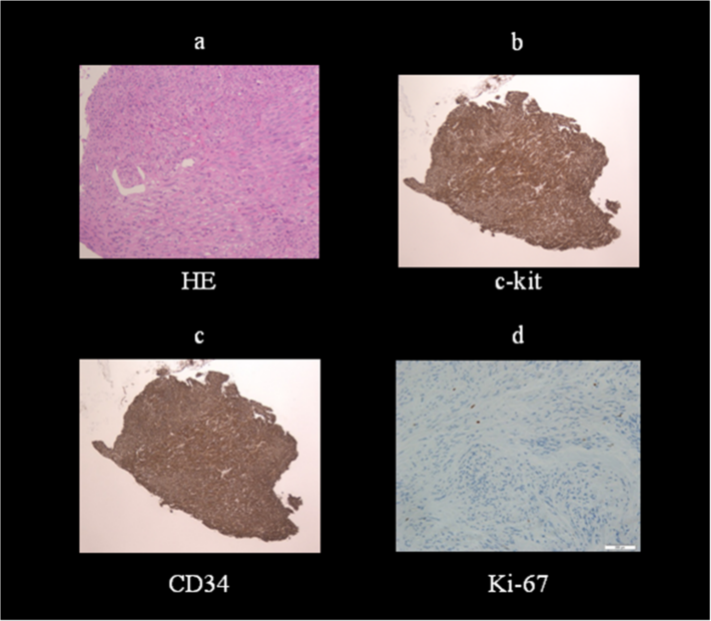
**Histopathological findings. a)** Spindle cell tumor **b**,**c)** Positive immunostaining for c-kit and CD34 **d)** <3% staining for Ki-67 antigen, a cellular proliferation marker.

Case 1 was a 46-year-old man. EGD revealed an SMT approximately 10 mm in diameter on the greater curvature of the antrum, which was smooth-sided and covered with normal mucosa (Figure 1). The lesion was tense, hard and immobile. We performed an endoscopic mucosal cutting biopsy (Figure 1). The histopathological findings revealed spindle cell tumor, with no mitotic figures. On the basis of positive immunostaining for c-kit and CD34 (Figure 2), a diagnosis of GIST was made. Furthermore, immunostaining of 3% for Ki-67 led to an assessment of a very low histological degree of malignancy. We then performed surgical resection in accordance with the GIST Therapeutic Guidelines.

## Discussion

Previously, GISTs were the most common submucosal tumors with potential malignancy in the upper gastrointestinal (GI) tract, no matter if their size is small or large, and it was difficult to predict their properties. Therefore, surgical excision is indicated if a histopathological diagnosis of GIST is made (GIST 2008). But the handling for 2 cm or less gastric GISTs does not have consensus in Japan. In the actual clinical site, the 2 cm or less gastric GISTs were resected in accordance with Japanese GIST Therapeutic Guidelines.

On the other hand, Suzuki et al (2010) reported that two of 16 cases in 2 cm or less GISTs increased during followed-up and recommended that it should be followed-up carefully. Also Nishida (2009) recommended a followed-up for 2 cm or less GISTs without ulceration and border irregularity. The recent rapid advances in endoscopic intervention therapy provide a potential method for *en bloc* resection of small Gastric SMTs. The modality of endoscopic treatment includes endoscopic band ligation (Liu-Ye et al. 2012; Sun et al. 2007), endpscopic submucosal dissection (ESD) (Lee et al. 2006; Filippo et al. 2012). The main defect of band ligation is that sloughed specimens are not available for pathological confirmation. Nevertheless, benign condition is comprised in SMTs like leopmyoma and heterotopicc pancreas, the problem is that perforation occurred during the ESD. Furthermore, successful complete resection of ESD is not 100%. In 2010, Bai et al (2010) reported that submucosal dissection technology for small GISTs < 2 cm in stomach is feasible with a 28% perforation rate, obviously higher than an overall 4% perforation in ESD for early gastric cancer. Also, full-thickness resection was reported treatment of SMTs. Zhou et al (2011) reported the complete resection rate was 100% in 26 patients with a SMT and there was no bleeding, peritonitis, and abdominal abscess. However, such procedures described previously cannot be accepted for a very benign condition in Japan.

We compared the two divided groups of GISTs with 2 cm or less and GISTs with >2 cm. The mean age was significantly higher in Group I than in Group II (*p <* 0.05). Therefore, GISTs were anticipated that the size increased as time passes. In tumor site, there was no GISTs >2 cm on the L site. Then, the GISTs on the M and U site were considered to have a chance of increasing. The histopathological and immunostaining findings from the surgically resected specimens were in agreement with those from the mucosal cutting biopsy specimens in all cases in Group I. In addition, all cases were in agreement because of the mitotic count. Therefore histological risk grade were also in agreement in all cases of Group I. These results indicate that a follow-up of 2 cm or less GISTs on the L site can also be considered acceptable. On the other hand, single case was not in agreement with the mitotic count between the mucosal cutting biopsy specimens and the surgically resected specimens in Group II. *En bloc* resection was very important for accurate histopathlogical diagnosis and histological risk grade of GIST. So traditionally, we consider that the gastric GISTs >2 cm and 2 cm or less gastric GISTs located on the M,U site were candidate for surgical treatment.

In conclusion, if 2 cm or less gastric SMTs located on the L site with a diagnosis of histpathlogical very low risk GIST, we consider that a follow-up of them can also be considered acceptable. On the other hand, traditionally, we consider that the gastric GISTs >2 cm and 2 cm or less gastric GISTs located on the M,U site were candidate for surgical treatment. This study demonstrated that the clinicopathological characteristic and clinical handling of the patients with 2 cm or less small gastric GISTs.

## Consent

Written informed consent was obtained from the patient for the publication of this report and any accompanying images.
